# A Cell-Based ELISA to Improve the Serological Analysis of Anti-SARS-CoV-2 IgG

**DOI:** 10.3390/v12111274

**Published:** 2020-11-08

**Authors:** Gianpaolo Zarletti, Massimo Tiberi, Veronica De Molfetta, Maurizio Bossù, Elisa Toppi, Paola Bossù, Giuseppe Scapigliati

**Affiliations:** 1 Dipartimento per l’Innovazione Biologica, Agroalimentare e Forestale, Università della Tuscia, 01000 Viterbo, Italy; gianpaolo.zarletti@gmail.com (G.Z.); massimo_tib@yahoo.it (M.T.); v.demolfetta@gmail.com (V.D.M.); 2 Department of Oral and Maxillofacial Science, “Sapienza” University, 00146 Roma, Italy; maurizio.bossu@uniroma1.it; 3 IRCCS Fondazione Santa Lucia, Experimental Neuropsychobiology Lab, Via del Fosso di Fiorano 64, 00143 Roma, Italy; e.toppi@hsantalucia.it (E.T.); p.bossu@hsantalucia.it (P.B.)

**Keywords:** SARS-CoV-2, COVID-19, in vitro IgG, B cell memory, cell-ELISA, spike S1 protein

## Abstract

Knowledge of the antibody-mediated immune response to SARS-CoV-2 is crucial to understand virus immunogenicity, establish seroprevalence, and determine whether subjects or recovered patients are at risk for infection/reinfection and would therefore benefit from vaccination. Here, we describe a novel and simple cell-ELISA specifically designed to measure viral spike S1-specific IgG produced in vitro by B cells in peripheral blood mononuclear cell (PBMC) cultures from a cohort of 45 asymptomatic (*n* = 24) and symptomatic (*n* = 21) individuals, with age ranging from 8 to 99 years. All subjects underwent ELISA serological screening twice, at the same time as the cell-ELISA (T2) as well as 35–60 days earlier (T1). Cryopreserved PBMCs of healthy donors obtained years before the COVID-19 pandemic were also included in the analysis. The preliminary results presented here show that out of 45 tested subjects, 16 individuals (35.5%) were positive to the cell-ELISA, 11 (24.5%) were concomitantly positive in the serological screening (T1 and/or T2), and only one person was exclusively positive in ELISA (T1) and negative in cell-ELISA, though values were close to the cutoff. Of note, five individuals (11.2%) tested negative in ELISA but positive in cell-ELISA and thus, they appear to have circulating B cells that produce antibodies against SARS-CoV-2, likely at levels that are undetectable in the serum, which challenges the negative results of the serological screening. The relative level of in vitro secreted IgG was measurable in positive subjects, ranging from 7 to 50 ng/well. Accordingly, all anti-SARS-CoV-2 antibody-positive subjects previously reported moderate to severe symptoms attributable to COVID-19, even though the RT-PCR data were rarely available to confirm viral infection. Overall, the described cell-ELISA might be an effective method for detecting subjects who encountered the virus in the past, and thus helpful to improve serological ELISA tests in the case of undetectable/equivocal circulating IgG levels, and a suitable and improved tool to better evaluate SARS-CoV-2-specific humoral immunity in the COVID-19 pandemic.

## 1. Introduction

Memory B cells and/or circulating plasma cells can be identified by their capability to secrete antigen-specific antibodies long after first encountering an antigen [1]; thus, they can provide important information related to past exposure. This feature has been employed to setup in vitro assays to detect and count antibody-secreting B cells through ELISPOT platforms [2]. With this method, antibody-producing cells secrete antibodies that bind to an antigen-coated plastic surface. The antigen–antibody complex is subsequently detected by a secondary antibody or substances covalently linked to enzyme/fluorochromes, and it is visualized as spots and can be counted using colorimetric or fluorescent methods [3]. ELISPOT has been successfully applied in vitro to measure the number of antibody-producing B cells, employing immobilized antigens from various pathogens including viral antigens [4,5]. Direct measurement of memory B-cells in peripheral blood can establish the magnitude and longevity of protection against infections and may be used in different fields of biomedical research, including epidemiology and vaccine development. We previously modified ELISPOT into a cell-based ELISA (cell-ELISA) that does not require the spot counting step, since the leukocytes are removed after incubation with the immobilized antigen, and the antigen-bound specific IgGs secreted in vitro by plasma cells are assayed by immunoenzymatic analysis [6]. Of note, quantification of B-cell secreted antibodies, if applicable in the clinical context, might be important for recognizing subjects who have encountered pathogens and identifying the occurrence of a disease. The latter is especially crucial during a pandemic, as in the case of the ongoing outbreak caused by the novel betacoronavirus named severe acute respiratory syndrome coronavirus-2 (SARS-CoV-2), first isolated in China in December 2019 and responsible for a highly transmissible disease named COVID-19. It is generally more severe in older age and is characterized by a range of mild respiratory symptoms that may advance to acute respiratory distress syndrome (ARDS) and multi-organ dysfunction [7]. Following host cell infection, SARS-CoV-2 triggers both innate and specific immune responses, but the precise immune pathways involved in activating the antivirus host defense mechanisms remain to be elucidated. To verify whether infection is ongoing, viral presence is diagnosed by analyzing the expression of viral genes via real-time PCR in biological material collected through nasopharyngeal swabs. The immune response can instead be measured by screening for the presence of specific antibodies in the blood using ELISA serological analyses [8]. The coronavirus envelope spike S1 protein is responsible for receptor binding and fusion to host cells, and it is involved in tropism and transmission capability [9]. The S1 protein has been shown to be a serological marker for COVID-19 [10]. Consequently, serological analyses via ELISA evaluate the presence of specific IgM/IgG by employing the recombinant spike protein S1 as adsorbed antigen. Antibody titers can be measured in intermediate and later stages of the infection (from day 7–10 onward) and are indicative of an antibody response [11]. The importance and robustness of ELISA systems in monitoring serum antibody response to the COVID-19 virus are confirmed by the number of diagnostic kit brands that are present on the market. However, a major limitation of ELISA-based platforms is circulating antibodies may become less and less detectable over time following vaccination or infection. On the other hand, circulating antigen-specific memory B cells can be present and persist long after immunization [4]. Upon reactivation, memory B cells are able to mount an antibody response decades after first encountering an antigen; hence, providing critical information regarding previous pathogen exposure. This feature is exploited by monitoring the humoral response of B cells, i.e., their capability to secrete in vitro specific IgG antibodies when restimulated with immunization antigen(s) [3].

A cell-based ELISA test adapted for COVID-19 would be useful to measure IgG specific for the S1 protein of SARS-CoV-2 produced in vitro by B cells, even when circulating antibodies become less detectable over time. Furthermore, by combining this assay with ELISA, it is possible to simultaneously detect the level of S1-specific plasma IgG and the presence of IgG-producing B cells in peripheral blood mononuclear cells (PBMCs) in plasma from the same donor. Such an approach may be helpful to improve serological analysis and determine the immunization status for SARS-CoV-2.

In this brief communication, we describe a new cell-ELISA, performed in combination with a commercially available COVID-19 serological test, in a cohort of 45 subjects. This assay is used to detect in vitro cell-produced IgG directed against the SARS-CoV-2 S1 protein. Although further work is required to confirm our preliminary results, we propose this cell-ELISA as a potential supplementary diagnostic tool useful for ruling out individuals who have very low serum IgG levels undetectable by ELISA, and thus improving the assessment of past infection and immune response in subjects during the COVID-19 pandemic.

## 2. Materials and Methods

### 2.1. Subjects Recruitment

Forty-five consecutive subjects (25 males and 20 females) of different ages (ranging from 8 to 99 years) undergoing COVID-19 serological analysis in an authorized diagnostic center (Centro Polispecialistico Giovanni Paolo I, Viterbo, Italy) were considered appropriate for enrollment in this study. The subjects of the study were consecutively enrolled in the study from the end of April to June 2020. The enrolled volunteers, who were asked to undergo serological tests and agreed to participate in the research project by signing an informed consent, were either asymptomatic or with mild to severe symptoms ascribable to COVID-19. The included subjects asked for ELISA (timepoint ELISA 1), either independently or after suggestion from their physician, in order to have a serology assessment of their infection status. All specimen and handling procedures were conducted taking precautions to provide a barrier between the specimen and personnel and using a class II biosafety cabinet (BSC). The following data were collected from each patient: age, sex, previous serology IgG screening, quantitative RT-PCR screening (nasopharyngeal swabs) when available, and presence of COVID-19 symptoms, as described in Table 1.

The blood was collected twice from each donor, at baseline (ELISA 1) and after 35–60 days (ELISA 2). In addition, pre-COVID-19 frozen PBMCs, used as controls, were obtained in 2017 from the blood of four healthy donors (Immunohematology and Transfusion Unit, Azienda Ospedaliera San Camillo-Forlanini) and cryopreserved in liquid nitrogen at Fondazione Santa Lucia LNS Cell Bank.

### 2.2. Ethics Approval

The study was approved by the Regional Ethical Board in Ospedale L. Spallanzani, Roma, (number 169, approval 22/07/2020) and, in accordance with the Helsinki Declaration, written informed consent was obtained from all subjects.

### 2.3. Cell-ELISA

The protocol was adapted from a previous study [5]. PBMCs came from donors whose plasma was already tested by ELISA 1 at least 35 days before. Cells were obtained by diluting whole blood (1:4) with saline physiological buffer (0.14 M NaCl), and 4 mL of diluted blood was loaded over 4 mL of a Lympholyte solution (Cedarlane, Burlington, Ontario, Canada) and centrifuged at 500× *g* for 20 min at room temperature. The PBMC layer was collected with a Pasteur pipette, and cells were washed with 10 mL of saline by centrifuging at 450× *g* for 10 min at room temperature. After washing, PBMCs were resuspended in 5 mL of RPMI medium containing 10% foetal calf serum FCS and antibiotics, and cells were counted in a hemocytometer with trypan blue. PBMCs collected in 2017 from healthy donors and cryoconserved in liquid nitrogen were thawed, resuspended in complete RPMI medium, and counted in trypan blue. The cell concentration was adjusted to 9 × 10^6^/mL, and 100 μL cell dilutions were plated into polystyrene wells pre-coated with recombinant spike S1 protein as antigen, supplied from a commercial ELISA kit (Dia.Pro Diagnostics Bioprobes srl, Milano, Italy. Lot 0420/5AA, ref: COV19G.CE.192). As controls, PBMCs were added to 10 μg/mL of cycloheximide (CXM, Sigma, St. Louis, MO, USA, cat C4859) to block protein synthesis, or plated in complete RPMI (110 μL/well) into a 96-well culture plate (Corning-Costar cat CLS3474, Sigma) to assess spontaneous release of IgG from PBMCs. Each experiment was conducted in duplicate. Cells were incubated for 48 h at 37 °C in a humidified incubator with occasional gentle shaking, and after incubation the cells were removed, and the wells washed three times with 150 μL of saline. The RPMI medium from the cells plated in the absence of antigen (spontaneous releases) was collected after centrifuging the plate at 450× *g* for 5 min and added (100 μL) to ELISA wells coated with antigen (Dia.Pro). The polystyrene wells were incubated for one hour at 25 °C and processed following the ELISA kit instructions and materials as described above. Briefly, the wells were washed three times with 150 μL of saline and incubated for one hour at 25 °C with a peroxidated anti-human IgG dilution contained in the kit, washed again as above, and enzyme reaction was visualized with a tetramethylbenzidine solution kit for 15 min at 25 °C. The reaction was then stopped with 3N sulfuric acid, and optical density (OD) was read at 450 nm in a plate reader. To evaluate the amount of IgG produced in vitro by cell-ELISA, human γ-globulins (Sigma G4386-5G) were dissolved in 50 mM carbonate-bicarbonate buffer (pH 9.4, from 100 μg/mL to 4 ng/mL) and were adsorbed overnight at 100 μL/well in duplicate wells of a polystyrene ELISA plate. The wells were then washed with Tris–HCl (50 mM, pH 7.4) containing 0.05% Tween-20 and 0.15 M NaCl (TTN). Binding sites were saturated with 3% bovine serum albumin in TTN for 30 min. After three washes with TTN, the wells were incubated with a peroxidated anti-human IgG antibody solution kit and processed as above.

### 2.4. Serology

Peripheral blood (5 mL) was collected in heparinized tubes from enrolled volunteers, and plasma was obtained by centrifugation at 490× *g* for 10 min. The levels of IgG antibody specific for the S1 protein of SARS-CoV-2 were measured in 10 μL of plasma diluted in 200 μL of binding solution using a commercial ELISA kit that employs the recombinant S1 peptide as adsorbed antigen (Dia.Pro). Specific anti-S1 IgGs were detected by incubation at 25 °C with a peroxidated goat anti-human IgG in 10 mM Tris-HCl (pH 6.8) for one hour, according to the manufacturer’s protocol. After washing with saline and adding the enzyme substrate (tetramethylbenzidine), color development proceeded for 15 min. The reaction was then stopped with 3 N sulfuric acid, and OD was read at 450 nm in a microplate reader. The ELISA kit contained an internal negative control (1% goat serum in 10 mM citrate buffer pH 6.0) and a positive control (IgG specific for SARS-CoV-2 in 10 mM citrate buffer pH 6.0 and 1% goat serum).

### 2.5. Statistical Analysis

Numerical data between different treatment groups were processed for their statistical significance by one-way ANOVA and two-tailed test of significance, with accepted *p*-values and T-values of <0.05 and >2.5, respectively.

## 3. Results

### 3.1. Cell-ELISA

PBMCs (9 × 10^5^ cells/well) from 45 subjects were tested by cell-ELISA, obtaining OD450 values ranging from a minimum of 0.004 to a maximum of 0.23. Net values were calculated for each sample by subtracting values obtained from adding the protein synthesis inhibitor CXM to cells. Spontaneous IgG release for each sample reached lower OD values than those obtained in the presence of CXM (not shown). As additional control, thawed PBMCs from four healthy control subjects, cryopreserved in 2017 (trypan blue viability > 90%), showed a mean OD450 value of 0.014 ± 0.01 when tested by cell-ELISA. A cutoff value for negative samples was arbitrarily established at an OD450 value of 0.065, calculated by adding two SDs on top of the mean OD450 of CXM samples (0.023 ± 0.021, N = 30). With this cutoff value, N = 16 samples were positive (with OD450 > 0.065), and N = 29 were negative.

**Figure 1:**
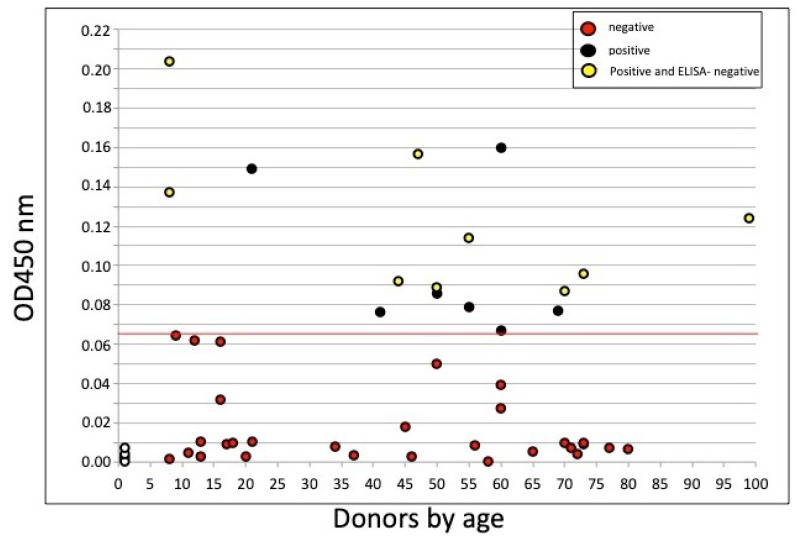
Cell-ELISA of in vitro-secreted IgG in peripheral blood mononuclear cells (PBMCs) from all donors reported with respect to age. The figure shows OD450 net values of 45 sampled donors, arbitrarily divided into negatives wgIith OD450 values below 0.065 (N = 29, red dots), and positives with OD450 values > 0.065 (N = 16). The OD450 values from pre-COVID-19 cryoconserved PBMCs (N = 4) are shown at an arbitrary 1 year age (empty dots). The cell-ELISA positive samples (N = 16) that obtained negative results in ELISA 2 (N = 9) are indicated as yellow dots. A red line indicates the cutoff OD450 value between negative and positive values.

The individual cell-ELISA values obtained from donors and presented as a function of age are shown in Figure 1, 16 samples out of 45 were positive (above the cutoff OD450 of 0.065, red line), with values ranging from 0.067 to 0.22, while 29 samples were negative at OD values < 0.065 (red dots), including PBMCs cryoconserved in 2017 (N = 4, empty dots). Notably, when individuals were concomitantly tested with cell-ELISA and ELISA (ELISA-2), among the 16 subjects tested positive by cell-ELISA, seven were also ELISA positive (43.7%, black dots), and nine tested negative (56.2%; yellow dots). Among the latter, likely considered as individuals who have encountered the virus, despite the negative serological results given by ELISA, two were children (<18 years), four were adults (18–55 years), and three were older (>55 years).

**Figure 2:**
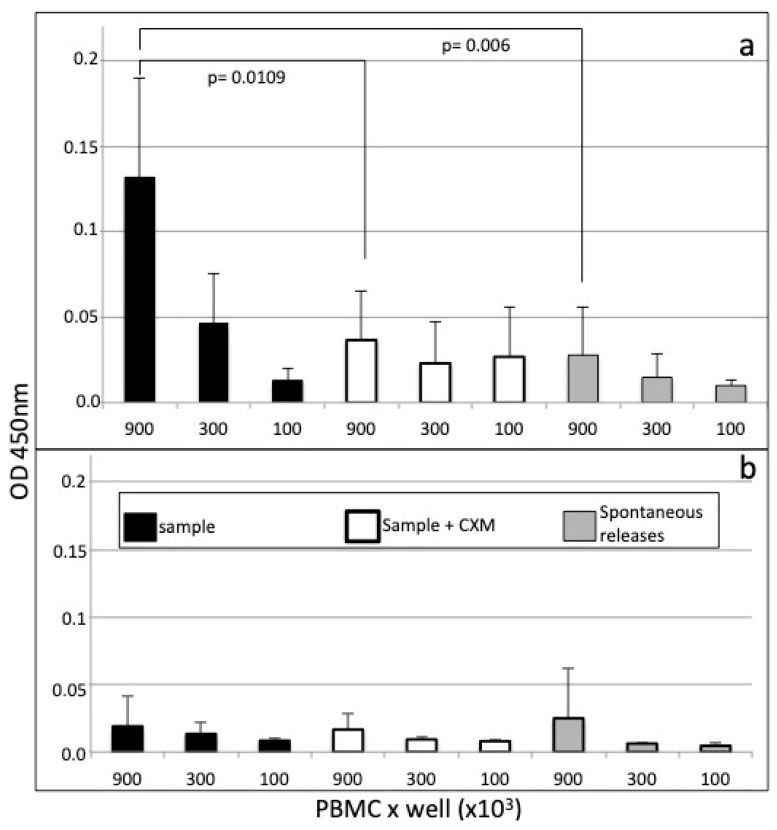
Cell-ELISA of in vitro-secreted IgG in PBMCs from 10 donors. The figure shows bars representing the mean ± SD net OD450 values of 5 positive (**a**) and 5 negative samples (**b**), obtained from indicated number of cells/well, incubated for 48 h in wells coated with spike S1 recombinant antigen (black bars, empty bars), or without antigen (grey bars). As control, the PBMCs were incubated with 10 μg/mL of cycloheximide to inhibit protein synthesis (empty bars) or incubated without antigen to assess spontaneous releases (grey bars). The treatment groups were analyzed by one-way ANOVA and two-tail test of significance, and significant differences are reported between indicated groups, with the indicated *p* value.

In vitro production of IgG anti-SARS-CoV-2 was determined in a subgroup of subjects (N = 10), where the optimal conditions for cell-ELISA in terms of PBMC number/well were set, as shown in Figure 2. Three dilutions of cells ranging from 9 × 10^5^ to 1 × 10^5^ per well were tested, and the mean OD450 results are shown for a subgroup of five positive and negative subjects (Figure 2a and Figure 2b, respectively). The mean ± SD OD450 values ranged from 0.131 ± 0.057 (9 × 10^5^ cells) to 0.047 ± 0.02 (3 × 10^5^ cells) and 0.013 ± 0.07 (1 × 10^5^ cells). With respect to the control PBMC cultures incubated with CXM, or to control cultures incubated without the antigen, the OD450 values were statistically significant (with *p* value < 0.05, two-tailed Student’s test, T value = 3.29) only when using the higher cell number of 9 × 10^5^ PBMC/well.

### 3.2. Combined ELISA/Cell-ELISA

To obtain information about the relationship between IgGs specific for the SARS-CoV-2 S1 protein present in the serum and those produced in vitro by memory B cells or plasma cells, we compared the data obtained by cell-ELISA with those by ELISA, and the results are shown in Table 2. The table summarizes data from the 16 donors who tested positive by cell-ELISA, who underwent complete serological analysis of IgG twice—a single ELISA (ELISA 1) at baseline and a second ELISA at least 35 days later (ELISA 2)—accompanied by cell-ELISA using serum and PBMCs from the same donor’s blood, respectively.

ELISA data are expressed as the ratio between the OD450 nm and cutoff values, as reported in the ELISA kit procedure (Dia.Pro). Cell-ELISA data are expressed as net OD450 values. The table shows the donors testing as negative (N = 5), equivocal (N = 1), and positive (N = 10) by ELISA 1 at day 0; the same donors tested as negative (N = 9), equivocal (N = 2), and positive (N = 5) by ELISA 2 after 35–60 days. By comparing serological and cell-ELISA data, Table 2 shows some donors were double-negative for both ELISA 1 and ELISA 2 (N = 5) but were positive by cell-ELISA; other donors positive by ELISA 1 were negative/equivocal in ELISA 2 (N = 5) but were indeed positive in cell-ELISA. Donors double-positive by both ELISA 1 and ELISA 2 (N = 4) were also positive in cell-ELISA. Only one person was exclusively positive in ELISA (T1) and negative in cell-ELISA, though with an OD450 value (0.064) close to the cutoff. To indirectly evaluate the amount of IgG produced in vitro using cell-ELISA, dilutions of human γ-globulins were evaluated by indirect ELISA. A reference curve was obtained (not shown), showing a linear trend from 360 ng/well to 10 ng/well, with R value = 0.94. With the reference curve, the relative deduced amounts of IgG produced in vitro by cell-ELISA are shown in Table 2, ranging from 7 ng to 50 ng per well (9 × 10^5^ cells).

## 4. Discussion

Accurate tests able to identify the SARS-CoV-2 virus and monitor the presence of antiviral antibodies are key in detecting those who have had an immune response to the virus, to help the management and surveillance of the virus, and thus to fight the COVID-19 pandemic. Diagnosis for SARS-CoV-2 is currently performed after running qRT-PCR of selected viral genes from nasal/respiratory tract swabs, monitoring the immune humoral response by serological assays, and/or measuring circulating IgM/IgG antibodies using various immunoenzymatic ELISA platforms available on the market. Previous studies have reported that serological testing may be useful to identify asymptomatic or subclinical infection with SARS-CoV-2 among medical workers in close contact with patients with COVID-19 [12]. However, in another study conducted on healthcare workers at risk for SARS-CoV-2 transmission, the majority of participants with positive IgG had a significant decline in antibody levels after one month [13]. In this context, information on the presence of memory B cells can be pivotal, since some studies have shown that circulating antibodies can shift to undetectable levels after vaccination or infection, while circulating antigen-specific memory B cells are still present [4,14,15]. In order to acquire information on the mounting of a B cell memory response for SARS-CoV-2, in this study, we adapted a simplified ELISPOT platform [5] to detect in vitro produced IgG for the spike S1 protein of SARS-CoV-2, which has already shown to be antigenic in humans and has been employed as an antigen in serological ELISAs [7]. The principle of this cell-ELISA is based on the use of PBMCs cultured in plastic wells pre-coated with recombinant S1 protein, as supplied in commercial ELISA kits. The cell-ELISA platform was tested with PBMCs obtained from donors whose plasma was already probed by ELISA 35–60 days earlier (ELISA 1). This timing was selected, taking into consideration recent data on the production of neutralizing antibodies at 39 days after infection [16,17], to discriminate between the reported half-life of IgG, being less than 30 days [18], and the presence of memory B cells and plasma cells, detected as early as 7 days after primary immunization [19], even in the absence of stimulatory factors (such as R848 and IL-2), as already reported for viral antigens [5].

Cell-ELISA positivity was assessed, using the arbitrary threshold of an OD450 value of 0.065 (as described above), on data obtained with PBMCs incubated with CXM, on spontaneous releases and from PBMCs cryoconserved before the COVID-19 pandemic.

Cell-ELISA data obtained for all tested samples, reported in Figure 1, showed 29 negatives and 16 positives. Data were reported in an age-dependent manner, where those tested positive by cell-ELISA (black dots) and negative by serological ELISA 2 (yellow dots) were indicated. Importantly, only one of the positive samples by ELISA 1 and ELISA 2 tested negative (at borderline) by cell-ELISA, and thus confirming the robustness of the assay. To our knowledge, this is the first description of an assay measuring IgG produced in vitro by B cells and specific for the SARS-CoV-2 S1 spike protein, an antigen shown to induce specific anti-COVID-19 IgG [10].

Although no conclusive statements can be drawn because of the relatively small size of the analyzed population, five individuals out of 45 (11.2%) tested negative by both ELISA1 and ELISA2 but positive by cell-ELISA, and thus, they appear to have circulating B cells that produce antibodies against SARS-CoV-2, likely at levels that are undetectable in the serum. This challenges the negative results from serological screening and suggests that the cell-ELISA may detect S1-specific IgG secreted by B cells even when a serological ELISA does not. This assumption is reinforced by the observation that all 16 subjects tested positive by cell-ELISA, including those who ELISA tested as negative and claimed to have had symptoms attributable to COVID-19.

This finding confirms previous observations describing that circulating antigen-specific memory B cells can be present and persist after immunization, even in the absence of detectable serum IgG [4]. In addition, although preliminary, our results further extend and improve recent findings on the kinetics of serum antibody responses in COVID-19 patients, where the ELISA for IgM/IgG was claimed to detect 75% of SARS-CoV-2-infected patients in the first week [20].

To confirm effective IgG production by B cells, in vitro cultures of PBMC were also evaluated in the presence of CXM, an antibiotic drug known to block initial phases of protein elongation in ribosomes, which have already been employed in a cell-ELISA animal model [5]. The data reported in Figure 2 suggest effective inhibition of protein synthesis and secretion by CXM and likely the blocking of IgG production by plasma cells. The same data also suggest the need to use a minimum concentration of 9 × 10^6^/mL PBMCs to gain reliable cell-ELISA data.

Even though the results we obtained by cell-ELISA appeared specific, as demonstrated by the CXM control, they were mainly based on low OD450 values. This probably is due to the small number of antibody-secreting cells present in peripheral blood and/or to the low sensitivity of reagents in the kit, which are calibrated for plasma/serum IgG. However, we choose to use a commercial kit to measure cell-secreted antiviral antibodies in order to support the enforceability of the assay in standard diagnostic labs. In addition, it is possible to select the antibody class to be tested by the secondary antibody employed.

To understand the possible levels of IgG secreted by PBMCs and the sensitivity threshold of the assay, the OD450 values of positive samples were compared to those from reference concentrations of human γ-globulin determined by ELISA. Obtained data suggest a minimal assay sensitivity of 7 ng IgG/well, which increased up to 50 ng/well of secreted IgG in donors. Interestingly, the donor whose PBMCs secreted the highest level of anti-S1 IgG in vitro tested negative in both serological ELISA 1 and ELISA 2 and declared no symptoms.

In conclusion, we propose here a modified ELISPOT platform, which was converted to a simpler cell-ELISA adapted to detect the S1 spike protein of SARS-CoV-2 and able to monitor the presence of memory B and plasma cells in the blood.

The proposed cell-ELISA, although needing more work addressed at increasing the number of donors and sampling over time, may improve upon conventional plasma or serum-based ELISAs, especially in the case of undetectable IgGs in serological analysis. Importantly, the assay could be of help in assessing the immunization status via S1 protein detection, and thus, providing a clearer picture of infection progression, and can readily be applied in clinical trials. Such important information may prove crucial in establishing seroprevalence in a more accurate way, in determining which individuals will benefit more than others from vaccination, and eventually in defining the efficacy of new vaccines.

## Figures and Tables

**Table 1:** Demographic and clinical characteristics of the recruited patients in the whole sample and by age group. The asymptomatics declared no symptoms attributable to COVID-19 before serology testing by ELISA. They were tested to obtain their anti-SARS-CoV-2 serological response as contacts of confirmed cases. The symptomatics declared either mild symptoms (one or more of the listed symptoms below) not requiring medical assistance, or severe symptoms and received medical assistance at home, or hospitalized. The main symptoms associated with COVID-19 were: fever or chills, cough, shortness of breath or difficulty breathing, fatigue, muscle or body aches, headache, new loss of taste or smell, sore throat, congestion or runny nose, nausea or vomiting, diarrhea.

	All Subjects	Age Group		
		<18	18–55	>55
Total *n*	45	11	16	18
Gender (male, *n*)	25	6	9	10
Asymptomatic (*n*)	24	6	7	11
Mild symptoms (*n*)	19	5	9	5
Severe symptoms * (*n*)	2	-	-	2
Rt-PCR (tested, *n*)	5	-	2	3
Rt-PCR (positive, *n*)	5		2	3
Serology IgG (tested, *n*)	45	11	16	18
Serology IgG ^$^ (positive, *n*)	12	0	7	4
Cell-ELISA (tested, *n*)	45	11	16	18
Cell-ELISA(positive, *n*)	16	2	8	6

* Hospitalized. ^$^ Serology testing was considered including both ELISA 1 and ELISA 2.

**Table 2:** ELISA/cell-ELISA comparison. The table shows data from 16 donors who tested positive by cell-ELISA for IgG with their age, and the plasma ELISA OD450 values for IgG measured at day 1 (ELISA 1), and in the same donor after 35–60 days (ELISA 2). A comparison between ELISA 2 results and cell-ELISA is shown, where a (+/+) indicates a positive result in both tests, and a (−/+) indicates a negative result in ELISA 2 and a positive result in cell-ELISA. The cell-ELISA numbers are the net OD450 values (°). The ELISA values are expressed as the ratio between OD450 of sample/cutoff value (*), considered negatives for <0.9, equivocal between 0.9–1.1, and considered positives for >1.1. A relative IgG concentration in cell-ELISA is indicated ^, as deduced from a reference γ-globulin ELISA.

Sample/Age	Day 0	Day 35–60			
	ELISA 1 *	ELISA 2 *	Cell-ELISA °	IgG ^ (ng/well)	ELISA2 vs. Cell-ELISA
#4, 60	4.3	1.8	0.16	35	+/+
#5, 60	5.5	3.3	0.066	7	+/+
#6, 55	3.6	1	0.079	9	−/+
#7, 50	4.2	2.1	0.085	10	++
#13, 55	3.5	0.43	0.114	16	−/+
#14, 41	0.19	0.16	0.076	8.5	−/+
#16, 8	0.18	0.17	0.203	50	−/+
#24, 24	3.64	1.51	0.092	11	+/+
#25, 69	4.77	4.21	0.077	8.5	+/+
#26, 50	3.01	0.88	0.088	10	−/+
#31, 21	4.96	1.02	0.149	32	−/+
#33, 8	0.16	0.20	0.137	30	−/+
#36, 99	0.23	0.23	0.124	18	−/+
#37, 73	1.21	0.74	0.096	11.5	−/+
#38, 70	1.05	0.26	0.087	10	−/+
#50, 47	0.33	0.18	0.157	34	−/+
